# Occurrence of Gastrointestinal Parasites in Fecal Samples from Free-Grazing Ducks in Northeastern Thailand

**DOI:** 10.3390/biology15191719

**Published:** 2026-09-30

**Authors:** Teputid Kuasit, Tongjit Thanchomnang, Jitsuda Kullawat, Sujittra Yahom, Siriporn Kuanamon, Wanchai Maleewong, Weerachai Saijuntha, Chairat Tantrawatpan

**Affiliations:** 1 Faculty of Medicine, Mahasarakham University, Maha Sarakham 44000, Thailand; teputid.k@msu.ac.th (T.K.); tongjit.t@msu.ac.th (T.T.); jitsuda.k@msu.ac.th (J.K.); sujitra.y@msu.ac.th (S.Y.); siriporn.ku@msu.ac.th (S.K.); 2 Biomedical Science Research Unit, Mahasarakham University, Maha Sarakham 44000, Thailand; 3 Department of Parasitology, Faculty of Medicine, Khon Kaen University, Khon Kaen 40002, Thailand; wanch_ma@kku.ac.th; 4 Mekong Health Science Research Institute, Khon Kaen University, Khon Kaen 40002, Thailand; 5 Division of Cell Biology, Department of Preclinical Sciences, Faculty of Medicine, and Center of Excellence in Stem Cell Research and Innovation, Thammasat University, Rangsit Campus, Pathum Thani 12120, Thailand

**Keywords:** helminth, zoonoses, poultry, intestinal parasites, avian host, One Health

## Abstract

Intestinal parasitic infections remain a persistent concern in tropical agricultural systems, where close interactions among animals, humans, and the environment facilitate parasite transmission. Free-grazing ducks, commonly raised in rice-field ecosystems in Thailand, may be exposed to a wide range of intestinal parasites. This cross-sectional study examined 312 fecal samples collected from five selected free-grazing duck flocks in northeastern Thailand from October to November 2025. All fecal samples were examined using the modified formalin–ethyl acetate concentration technique. Parasites were identified based on egg and larval morphology. Positive parasite detections were found in 168 samples, yielding an overall percentage of positive fecal samples (%PFS) of 53.8%. The %PFS across the five provinces ranged from 42.5% to 63.5%. Among the positive fecal samples, the most frequently detected parasites were unidentified nematodes (38.1%), trichurid-like eggs (30.3%), strongyle-type eggs (25.0%), echinostome-like eggs (21.4%), and *Ascaridia* spp. (18.4%). Among the positive samples, small operculated trematode (SOT) and hymenolepidid-like eggs were detected in 3.0% and 1.2%, respectively; however, these findings may represent spurious passage. Single morphotype detection predominated, while co-detection of multiple morphotypes occurred less frequently. These findings document a diverse assemblage of intestinal parasite morphotypes in fecal samples from free-grazing ducks in northeastern Thailand, potentially reflecting exposure through aquatic and soil-associated routes.

## Simple Summary

Free-grazing ducks forage in rice fields in northeastern Thailand, where they may encounter a range of parasites. We examined fecal samples from five selected duck flocks and detected parasite eggs or larvae in approximately half of the samples. The detected forms included morphotypes resembling those of trematodes, nematodes, and cestodes, but most could not be identified to species from their appearance alone. Exposure to soil-associated sources or aquatic intermediate hosts is biologically plausible in rice-field environments; however, this study did not investigate how the parasites were acquired or whether the ducks were infected by every parasite detected. Further molecular and epidemiological studies are needed to establish species identities, clarify the role of ducks as hosts, and assess any veterinary or public health significance.

## 1. Introduction

Intestinal parasitic infections remain a major global public health challenge, particularly in tropical and subtropical regions where environmental conditions favor parasite transmission. The World Health Organization estimates that more than 1.5 billion people are infected with soil-transmitted helminths [1], while more than 40 million people are infected with foodborne trematodes worldwide [2]. In Thailand, national surveys have reported persistent helminth infections, especially in rural areas of the northeastern region, where prevalence varies widely depending on locality and population group [3,4,5]. Environmental sanitation, traditional dietary practices, and close interactions between humans, livestock, and aquatic ecosystems contribute to sustained transmission cycles.

Free-grazing duck (*Anas platyrhynchos domesticus*) farming represents a common agricultural practice in Thailand, particularly in rice-producing regions. After harvest, flocks of ducks are released into rice paddies where they forage on leftover grains, insects, freshwater snails, fish, tadpoles, and other aquatic organisms [6]. While this system offers economic benefits and natural pest control, it also creates ecological conditions that are favorable for parasite transmission. Many aquatic organisms consumed by ducks serve as intermediate hosts for trematodes and other helminths, facilitating the completion of complex life cycles within rice-field environments [6,7]. Previous research in Thailand has demonstrated a high prevalence of zoonotic echinostome infections in free-grazing ducks, highlighting their role as reservoir hosts within agricultural transmission systems [6].

Ducks may also harbor soil-transmitted helminths and other intestinal parasites. Eggs or larvae of nematodes such as trichurids and ascarids may survive or develop under warm, humid environmental conditions, potentially increasing parasite exposure in free-range production systems [8]. Studies from Bangladesh, Nepal, Vietnam, and Indonesia have also shown diverse helminth communities in free-grazing or semi-intensive duck production systems, suggesting that free-range husbandry may enhance parasite transmission through continuous environmental exposure [7,9,10,11,12]. However, the zoonotic potential of the soil-transmitted parasites detected in ducks has not been confirmed, particularly because identification based solely on egg morphology generally cannot reliably distinguish avian parasites from morphologically similar zoonotic species.

Across many Asian countries, including Thailand, agricultural landscapes are characterized by extensive rice cultivation, seasonal flooding, and the widespread practice of integrated rice–duck farming systems [13]. This ecological setting supports both aquatic and soil-mediated parasite transmission cycles and creates a close interface between animals, humans, and the environment. Despite this, most previous studies in Thailand have focused on specific parasite groups, particularly zoonotic trematodes [14,15,16]. Comprehensive data on the broader spectrum of intestinal parasites in free-grazing ducks in northeastern Thailand remain limited.

This study describes the intestinal parasite morphotypes detected in fecal samples from five selected free-grazing duck flocks in northeastern Thailand, including their frequency of detection and co-detection within samples. The findings provide baseline information for these flocks and help identify morphotypes that warrant species-level identification and further investigation of possible transmission routes in rice-field ecosystems.

## 2. Materials and Methods

### 2.1. Sample Collection

**Figure 1:**
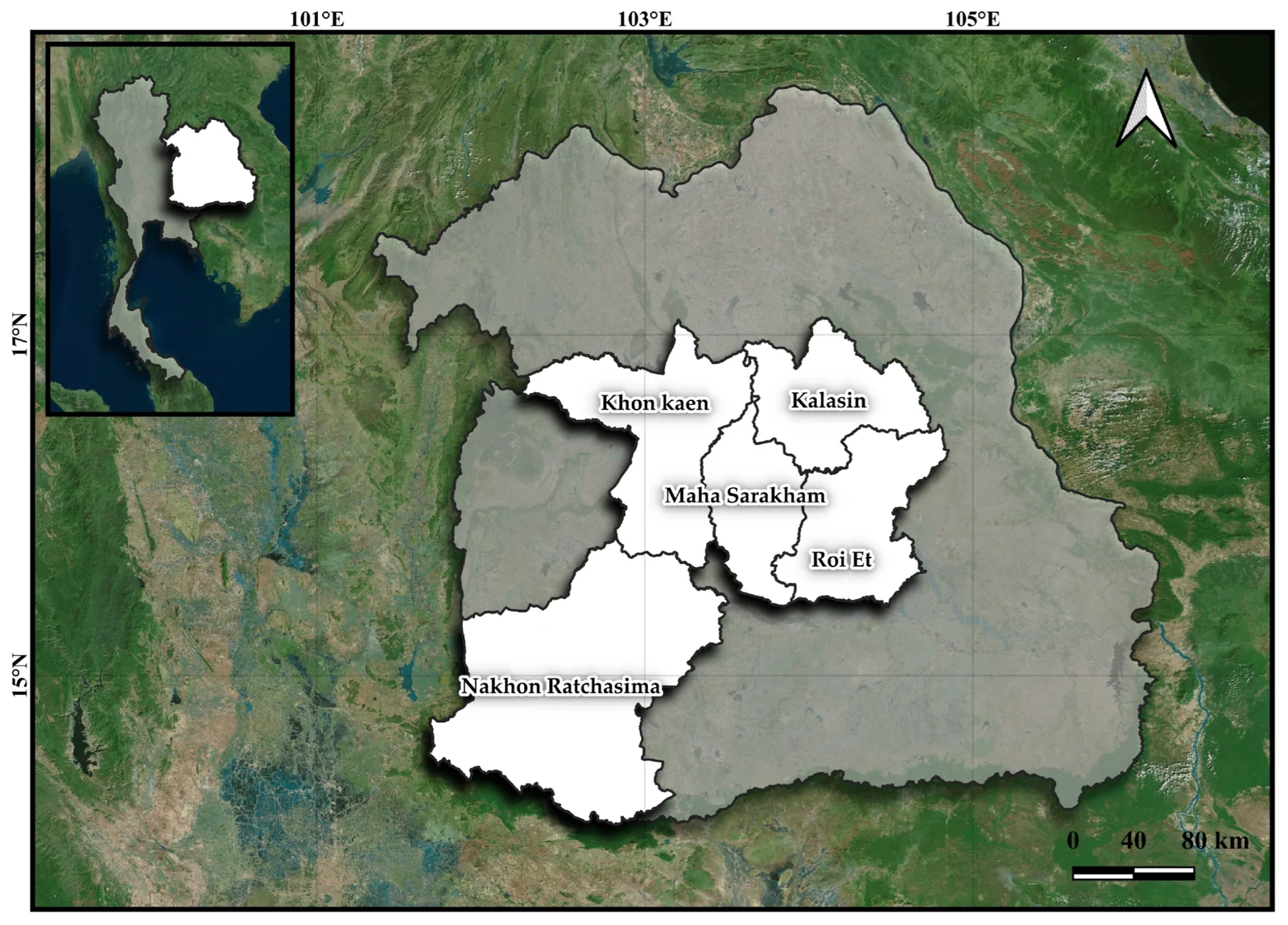
Map showing the geographical distribution of sampled free-grazing ducks across five provinces in northeastern Thailand. The sampling location map was generated using the free and open-source QGIS Long Term Release (LTR) version 3.44 “https://qgis.org/ (accessed on 15 August 2026)”. Provincial boundary shapefiles of Thailand were obtained from the Open Development Mekong platform and used as the base map for displaying the sampling locations “https://data.thailand.opendevelopmentmekong.net/th/dataset/thailand-provincial-boundaries” (accessed on 15 August 2026).

A cross-sectional study was conducted from October 2025 to November 2025 among five free-grazing duck flocks, one from each of five provinces in northeastern Thailand: Kalasin, Maha Sarakham, Khon Kaen, Roi Et, and Nakhon Ratchasima (Figure 1 and Table 1). Fresh fecal samples were collected from the ground in the duck pen shortly after the free-grazing flocks were released into the rice fields in the morning. Fresh, intact, spatially separated fecal deposits were selected to reduce the likelihood of sampling the same duck more than once. Approximately 5–10 g of feces was collected from the inner portion of each fecal sample using a sterile spatula to minimize soil contamination. The sample was then placed in a sterile container and transported in a cooled box (on ice) to the Faculty of Medicine, Mahasarakham University, for further examination.

### 2.2. Parasitological and Statistical Analyses

The modified formalin–ethyl acetate concentration technique (FECT) was used [17]. All fecal samples were processed within 24 h after collection. Approximately 2 g of feces were emulsified in 7 mL of 10% formalin (Thermo Fisher Scientific, Taren Point, Australia) and filtered through gauze (Zhende Medical, Shaoxing, China), centrifuged at 600× *g* for 5 min, the supernatant was discarded, and then the remainder mixed with 3 mL of ethyl acetate and centrifuged again at 600× *g* for 5 min. The upper layers were discarded, and the remaining sediment was resuspended in 2 mL of 10% formalin and stored at room temperature until microscopic examination. The preserved sediments were kept at room temperature and examined microscopically within 24 h of preparation. In total, 312 sediment preparations (one per sample) were examined after mixing two drops (~120 μL) of sediment with iodine solution (Ajax Finechem, New South Wales, Australia). Five microscopists (T.K., T.T., J.K., S.Y. and W.S.) who had received intensive training in the identification of parasite eggs and larvae examined the samples using an Olympus CX31 light microscope (Olympus Corporation, Tokyo, Japan) at 100× and 400× magnification. Parasites were identified to the genus or group level, where possible, based on the morphology of their eggs and larvae [18,19]. Species-level identification was generally not attempted because most observed eggs and larvae lacked sufficient diagnostic features for reliable identification. For quality control, all suspected parasite eggs and larvae were photographed at 400× magnification using an Olympus SC180 digital camera (Olympus Corporation, Tokyo, Japan). The images were subsequently reviewed to reassess the morphological identifications, and egg and larval dimensions were measured using ImageJ version 1.54 [20].

The number of samples collected from each flock varied according to the number of suitable fresh deposits available at the time of sampling. Ducks were not individually marked, so multiple samples from the same duck could not be ruled out. The fecal sample was therefore the unit of analysis, and results are reported as the percentage of positive fecal samples (%PFS), rather than the prevalence of infection in individual ducks. Information on anthelmintic treatment, detailed flock management, contact with other animals, and flock movement was unavailable; therefore, their possible contributions to differences in morphotype detection among the five flocks could not be assessed.

## 3. Results

A total of 312 fecal samples collected from five free-grazing duck flocks in northeastern Thailand were examined for intestinal parasites. Of these, 168 were positive, corresponding to an overall %PFS of 53.8%. The %PFS among the five sampled flocks ranged from 42.5% to 63.5%. The highest value was recorded in the Roi Et flock (63.5%; 54/85), followed by the Maha Sarakham (53.9%; 41/76), Khon Kaen (50.8%; 32/63), Nakhon Ratchasima (50.0%; 24/48), and Kalasin (42.5%; 17/40) flocks (Table 2).

**Figure 2:**
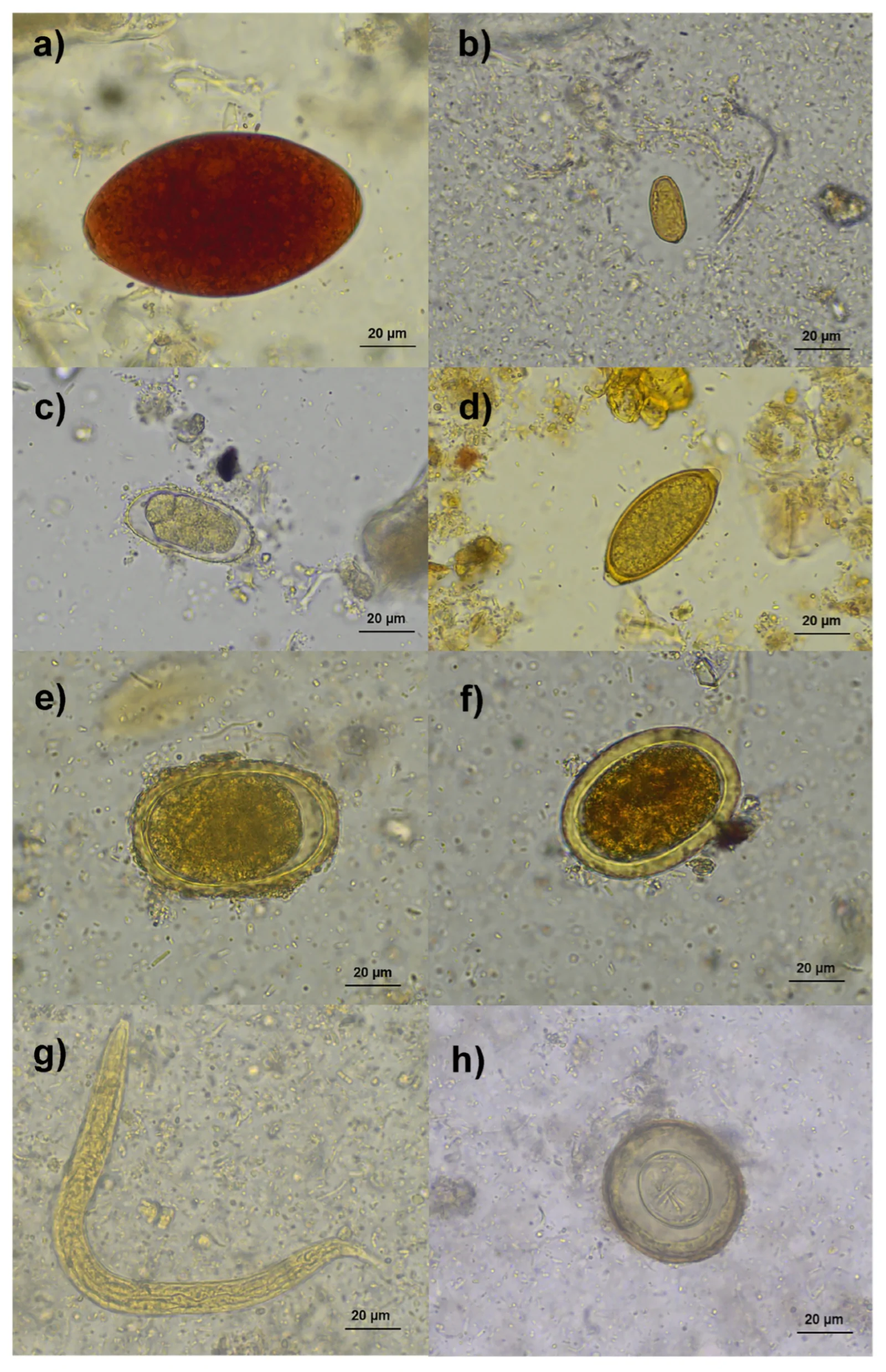
Microscopic images of helminth stages detected in fecal samples from free-grazing ducks. (**a**) echinostome-like egg; (**b**) small operculated trematode egg; (**c**) strongyle-type egg; (**d**) trichurid-like egg; (**e**) *Ascaridia* sp. fertilized egg; (**f**) *Ascaridia* sp. decorticated egg; (**g**) unidentified nematode larva; (**h**) hymenolepidid-like egg. All images were captured under light microscopy at 400× magnification using iodine staining.

Seven morphotypes of intestinal parasites were identified based on egg and larval morphology (Figure 2). Dimensions of all morphotypes were measured and shown in Table S1. Among the 168 positive fecal samples, the most frequently detected parasites were unidentified nematodes (38.1%) and trichurid-like egg (30.3%), followed by strongyle-type egg (25.0%), echinostome-like egg (21.4%), and *Ascaridia* spp. (18.4%). Small operculated trematode (SOT) and hymenolepidid-like egg were detected at 3.0% and 1.2%, respectively. Among the sampled flocks, echinostome-like eggs were detected most frequently in fecal samples from the Maha Sarakham and Nakhon Ratchasima flocks, whereas soil-transmitted helminths, particularly unidentified nematodes, trichurid eggs, and *Ascaridia* spp., were detected more frequently in samples from the Roi Et and Khon Kaen flocks. The percentages of individual parasite taxa exceeded 100% within a sampling locality because some fecal samples contained more than one morphotype (Table 3).

Analysis of parasite co-detection showed that a single morphotype predominated, occurring in 72.0% (121/168) of positive fecal samples (Table 3). Two morphotypes were detected in 22.0% (37/168) of positive samples, whereas three and four or more morphotypes were each detected in 3.0% (5/168). Among the sampled flocks, fecal samples from the Roi Et flock had the highest frequency of multiple morphotype detections, while positive samples from the Khon Kaen and Kalasin flocks predominantly contained a single morphotype. The sample-level dataset is provided in Table S2.

## 4. Discussion

This study provides updated evidence that intestinal parasites are commonly detected in fecal samples from free-grazing duck flocks in northeastern Thailand. Because only one flock was sampled in each province, the findings are descriptive and should not be interpreted as evidence of province-level patterns or differences. The overall %PFS was 53.8%, ranging from 42.5% to 63.5% across the five sampled flocks. These findings suggest that rice-field–wetland agroecosystems may provide favorable conditions for parasite transmission and frequent exposure among free-grazing ducks at the selected localities. Similar findings have been reported in Thailand and other Asian countries, indicating that ecological conditions and husbandry practices associated with free-grazing systems may facilitate exposure to various intestinal parasites [6,7,9,10,12].

The findings should also be interpreted in light of the diagnostic limitations of this study. Parasite detection relied on a single qualitative examination using the FECT. Although FECT can improve the recovery of helminth eggs and larvae compared with unconcentrated preparations, its sensitivity varies among parasite taxa and may be reduced in low-intensity infections [21,22]. Detection may also be influenced by the amount of fecal material examined, the distribution of diagnostic stages within the sample, and temporal variation in egg or larval shedding. Furthermore, the use of only one fecal sample and one diagnostic technique may have resulted in some infections being missed and, consequently, an underestimation of parasite occurrence and diversity [22,23]. Therefore, a negative result indicates only that no parasite stage was detected using the method applied and does not exclude infection. Future studies should consider repeated sampling and complementary diagnostic approaches, such as quantitative egg-counting, flotation or sedimentation techniques, and molecular assays, to improve diagnostic sensitivity and taxonomic resolution.

The diversity of parasite taxa detected reflects the ecological complexity of these production systems. The detection of eggs morphologically consistent with echinostome-like trematodes and SOTs suggests that free-grazing ducks may be exposed to aquatic intermediate hosts while foraging in flooded rice fields. Freshwater snails and other aquatic organisms serve as intermediate hosts for many trematodes and contribute to the maintenance of their life cycles in rice-field ecosystems [15,24]. Several snail groups, including lymnaeids, viviparids, bithyniids, ampullariids, and turritellids, are known to facilitate parasite transmission in such environments [25,26]. The relatively high occurrence of echinostome-like eggs, including co-detection of multiple morphotypes, further highlights the importance of aquatic exposure routes inferred from known life cycles and supports previous findings that free-grazing ducks can act as reservoir hosts in Southeast Asia [6,27,28].

In parallel, the detection of soil-transmitted helminths, including trichurid, ascarid, and strongyle-type nematodes, indicates frequent contact with contaminated soil in warm, humid environments where infective stages can persist [8]. The occurrence of parasites with aquatic and soil-associated life cycles may partly explain the diverse parasite assemblage observed, indicating that rice-based farming systems provide suitable conditions for multiple transmission pathways [29]. Other parasites, such as SOT and hymenolepidid-like egg, were detected at low %PFS but reflect complex trophic transmission pathways involving invertebrate and vertebrate hosts. The hymenolepidid-like eggs resemble those of *Hymenolepis diminuta* by having oval shape, and containing a six-hooked oncosphere without polar filaments. This cestode primarily infects rodents and requires an arthropod intermediate host to complete its life cycle [30]. The presence of hymenolepidid-like eggs, as well as SOT eggs, in duck feces may represent spurious passage following the ingestion of rodent feces or other egg-contaminated material in rice fields rather than patent infection or sustained transmission in ducks. Nevertheless, these findings suggest ecological interactions among rodents, arthropods, and domestic animals within rice-field agroecosystems.

Moreover, the unidentified nematode larvae may have originated from eggs that hatched before sample collection or from free-living nematodes introduced through environmental contamination because the fecal samples were collected from the ground. Although only fresh, intact fecal deposits were selected, transported on ice in a cool box, and processed within 24 h, environmental contamination or larval hatching before collection cannot be completely excluded [31,32]. Therefore, the presence of these larvae should not be interpreted as evidence of patent infection or confirmation that ducks served as definitive hosts; molecular identification or examination of adult nematodes would be required to determine their taxonomic identity and origin.

Single-morphotype detection predominated, although co-detection of multiple morphotypes was also observed, consistent with previous studies in free-grazing ducks [9,33]. Co-detection of multiple morphotypes may influence host immunity and parasite transmission dynamics, thereby increasing epidemiological complexity. The higher parasite diversity observed in some localities may reflect localized environmental or management factors, such as prolonged exposure to flooded habitats or higher densities of intermediate hosts. Overall, parasite diversity is likely shaped by environmental factors such as elevation, seasonality, and local geography, which influence the distribution of intermediate hosts [34,35]. However, this study used a qualitative diagnostic approach without quantifying infection intensity. Therefore, the results indicate only the presence of parasite morphotypes and cannot confirm infection burden, species identity, site of infection, or whether ducks served as definitive, accidental, or mechanical hosts.

Importantly, several parasite groups identified in this study belong to taxa of recognized zoonotic importance, especially echinostome-like egg [6]. Previous studies have demonstrated substantial genetic diversity and cryptic species within trematodes and their snail hosts in this region, suggesting that morphologically similar parasites may represent distinct transmission units [36]. In rice-farming systems, interactions among ducks, other animals, humans, and aquatic environments may support the life cycles of diverse parasites. However, the zoonotic potential of the parasite morphotypes detected in this study could not be determined from fecal morphology alone. Molecular characterization is therefore required to identify these parasites at the species level and reliably assess their zoonotic potential [37].

Taken together, these findings document the occurrence of diverse parasite morphotypes in duck fecal samples collected from rice-field environments. Their value is particularly relevant in regions where flocks forage over large areas and routine veterinary surveillance is limited. Nevertheless, fecal microscopy cannot establish species identity, host competence, or direct transmission to humans; the zoonotic significance of the detected groups should therefore not be inferred without examination of adult parasites or molecular confirmation. The fecal sediment in this study was preserved in formalin. Because formalin fixation can cause DNA fragmentation and cross-linking, potentially reducing DNA recovery and PCR amplification success [38], PCR was not attempted on these samples. Molecular analysis of formalin-preserved material may nevertheless be feasible under some conditions. Multi-country studies combining species-level diagnostics, quantitative egg counts, flock movement, seasonality, and intermediate-host sampling, including molecular identification, would enable more robust comparisons and clarify which production practices consistently predict infection.

## 5. Conclusions

This study documented seven parasite morphotypes, unidentified nematode larvae, trichurid-like eggs, *Ascaridia* spp., strongyle-type eggs, echinostome-like eggs, small operculated trematode eggs, and hymenolepidid-like eggs, in fecal samples from five selected free-grazing duck flocks in northeastern Thailand. Some morphotypes were consistent with parasites commonly occurring in the gastrointestinal tract of ducks, whereas others may have resulted from incidental infection, environmental contamination, or spurious passage. Although some detected groups include zoonotic species, their zoonotic importance could not be established by fecal morphology alone. To our knowledge, this is the first multi-site survey to describe the diversity and co-detection patterns of gastrointestinal parasite morphotypes in fecal samples from free-grazing duck flocks associated with rice-field environments across northeastern Thailand. Molecular identification, longitudinal sampling, and investigations of adult parasites, intermediate hosts, environmental sources, flock movement, and management practices are needed to clarify parasite identity, host status, transmission pathways, and veterinary or public health significance.

## Figures and Tables

**Table 1:** Locations of five selected free-grazing duck flocks in northeastern Thailand.

Code	District	Province	Flock Size *	Foraging Habitat	Date of Collection	Coordinate
KK	Phon	Khon Kaen	~150–180	Rice field	15 October 2025	15.807° N/102.534° E
KS	Na Mon	Kalasin	~180–200	Rice field	21 October 2025	16.556° N/103.775° E
MS	Kantharawichai	Maha Sarakham	~230–250	Rice field	2 October 2025	16.271° N/103.218° E
NR	Sida	Nakhon Ratchasima	~160–200	Rice field	18 November 2025	15.630° N/102.603° E
RE	Mueang Roi Et	Roi Et	~280–300	Rice field	3 November 2025	16.045° N/103.591° E

* Number of ducks per flock.

**Table 2:** Percentage of fecal samples positive for gastrointestinal parasites among five selected free-grazing duck flocks in northeastern Thailand.

Locality	*N* *	Number of Positive Fecal Samples (%)	Number of Fecal Samples with Each Morphotype of Parasite (%) **						
			Un	Tr	As	St	Ec	SOT	Hd
Kalasin	40	17 (42.5)	3 (17.6)	4 (23.5)	0 (0.0)	11 (64.7)	1 (5.9)	0 (0.0)	0 (0.0)
Maha Sarakham	76	41 (53.9)	13 (31.7)	6 (14.6)	1 (2.4)	3 (7.3)	26 (63.4)	2 (4.9)	2 (4.9)
Khon Kaen	63	32 (50.8)	11 (34.4)	13 (40.6)	0 (0.0)	8 (25.0)	2 (6.2)	0 (0.0)	0 (0.0)
Roi Et	85	54 (63.5)	22 (40.7)	21 (38.9)	29 (53.7)	16 (29.6)	0 (0.0)	3 (5.5)	0 (0.0)
Nakhon Ratchasima	48	24 (50.0)	15 (62.5)	7 (29.2)	1 (4.2)	4 (16.7)	7 (29.2)	0 (0.0)	0 (0.0)
Total	312	168 (53.8)	64 (38.1)	51 (30.3)	31 (18.4)	42 (25.0)	36 (21.4)	5 (3.0)	2 (1.2)

* Number of fecal samples examined; ** Percentages for each parasite morphotype were calculated using all parasite-positive fecal samples of each locality as the denominator. Morphotype of parasites: Un, unidentified nematodes; Tr, trichurid-like egg; As, *Ascaridia* spp.; St, strongyle-type egg; Ec, echinostome-like egg; SOT, small operculated trematode egg; Hd, hymenolepidid-like egg.

**Table 3:** Distribution of single and multiple morphotype detections among 168 positive duck fecal samples from five selected flocks in northeastern Thailand.

Parasite Detection Categories		KS	MS	KK	RE	NR	Total
Single morphotype	Unidentified nematodes (Un)	3	5	9	6	7	30
	Trichurid-like egg (Tr)	3	5	13	7	2	30
	*Ascaridia* spp. (As)	0	1	0	11	0	12
	Strongyle-type egg (St)	10	1	6	6	0	23
	Echinostome-like egg (Ec)	0	18	2	0	5	25
	Small operculated trematode egg (SOT)	0	1	0	0	0	1
	Hymenolepidid-like egg (Hd)	0	1	0	0	0	1
	Subtotal	16	32	30	29	14	121
Multiple morphotypes	Un + Tr	0	0	0	3	4	7
Two morphotypes	Un + As	0	0	0	5	0	5
	Un + Ec	0	6	0	0	1	7
	Un + St	0	1	2	1	3	7
	Tr + As	0	0	0	6	1	7
	Tr + St	1	0	0	0	0	1
	Tr + Ec	0	1	0	0	0	1
	St + Ec	1	0	0	0	1	2
	Subtotal	2	8	2	15	10	37
Three morphotypes	Un + St + SOT	0	0	0	1	0	1
	Un + As + St	0	0	0	1	0	1
	Un + St + Tr	0	0	0	1	0	1
	Un + St + Hd	0	1	0	0	0	1
	Tr + As + St	0	0	0	1	0	1
	Subtotal	0	1	0	4	0	5
Four or more morphotypes	Un + As + St + SOT	0	0	0	1	0	1
	Un + As + Tr + St	0	0	0	2	0	2
	Un + St + Ec + SOT	0	1	0	0	0	1
	Un + As + Tr + St + SOT	0	0	0	1	0	1
	**Subtotal**	**0**	**1**	**0**	**4**	**0**	**5**

Province name: KS; Kalasin, MS; Maha Sarakham, KK; Khon Kaen, RE; Roi Et, NR; Nakhon Ratchasima.

## Data Availability

The original contributions presented in the study are included in the article/Supplementary Materials. Further inquiries can be directed to the corresponding authors.

## Supplementary Materials

The following supporting information can be downloaded at: https://www.mdpi.com/article/10.3390/biology15191719/s1. Table S1: Morphometric measurements of parasite eggs and larvae detected in fecal samples from free-grazing ducks in northeastern Thailand. Table S2. Parasitic infections detected from duck fecal samples.

## Abbreviations

The following abbreviations are used in this manuscript:

DNA	deoxyribonucleic acid
FECT	formalin–ethyl acetate concentration technique
g	gram
× *g*	relative centrifugal force
h	hour
min	minutes
mL	milliliter
PCR	polymerase chain reaction
%PFS	percentage of positive fecal samples
SOT	small operculated trematode
μL	microliters
