# Supplementary material for: Occurrence of Gastrointestinal Parasites in Fecal Samples from Free-Grazing Ducks in Northeastern Thailand
Source: Biology (Basel). 2026 Sep 30;15(19):1719. doi: 10.3390/biology15191719 (PMC13663500; doi:10.3390/biology15191719)
Supplement: Supplementary file 1 [file biology-15-01719-s001.zip › Table S1.pdf]

Table S1. Morphometric measurements of parasite eggs and larvae detected in fecal samples from free-grazing ducks in northeastern Thailand.

Parasite morphotype	Developmental stage	*n*	Length (µm)	Width (µm)
Ascaridia spp.	egg	6	69.552–82.963	45.946–54.433
Echinostomes-like trematode	egg	7	95.038–105.281	61.340–63.649
Hymenolepidid-like cestode	egg	2	56.750–57.487	50.142–50.544
Small operculated trematode	egg	3	23.281–25.835	12.962–14.339
Strongyle-type nematode	egg	6	48.120–57.069	29.324–38.189
Trichurid-like nematode	egg	4	25.792–26.667	55.410–59.342
Unidentified nematode	larva	5	222.018–343.39	13.557–19.448
